# Long-term trends of tick-borne pathogens in regard to small mammal and tick populations from Saxony, Germany

**DOI:** 10.1186/s13071-019-3382-2

**Published:** 2019-03-26

**Authors:** Daniel Galfsky, Nina Król, Martin Pfeffer, Anna Obiegala

**Affiliations:** Institute of Animal Hygiene and Veterinary Public Health, University of Leipzig, An den Tierkliniken 1, 04103 Leipzig, Germany

**Keywords:** Rodent, *Myodes glareolus*, *Apodemus flavicollis*, *Bartonella*, *Rickettsia*, *Hepatozoon*, *Neoehrlichia*, *Anaplasma phagocytophilum*, Ticks, *Ixodes ricinus*

## Abstract

**Background:**

Rodents are important in the life-cycle of ticks as hosts for immature developmental stages. Both rodents and ticks are of public health interest as they are reservoirs and vectors for different tick-borne pathogens (TBP). The aim of this study was to reassess the prevalence of TBP in previously studied areas of the city of Leipzig (Saxony, Germany).

**Methods:**

In the years 2015–2017 rodents and ticks were collected in parks and forest areas in Saxony. DNA was extracted from the rodents, attached and questing ticks. Samples were screened for the presence of *Anaplasma phagocytophilum*, *Babesia* spp., *Borrelia burgdorferi* (*s.l.*), “*Candidatus* Neoehrlichia mikurensis” (CNM), *Bartonella* spp., *Hepatozoon* spp. and *Rickettsia* spp. using PCR methods. Rodent, attached nymph and questing tick (nymph and adult) samples were tested individually, while attached larvae were further processed in pools.

**Results:**

A total of 165 rodents (*Apodemus agrarius*, *n *= 1; *A. flavicollis*, *n *= 59; *Arvicola terrestris*, *n *= 1; *Myodes glareolus*, *n *= 104), 1256 attached ticks (*Ixodes ricinus*, *n *= 1164; *Dermacentor reticulatus*, *n *= 92) and 577 questing ticks (*I. ricinus*, *n *= 547; *D. reticulatus*, *n *= 30) were collected. The prevalence levels in rodents were 78.2% for *Bartonella* spp., 58.2% for CNM, 49.1% for *B. burgdorferi* (*s.l.*) 29.1% for *Rickettsia* spp. and 24.2% for *Hepatozoon* spp. The minimal infection rates (MIR) in attached larvae ticks were 39.8% for *Rickettsia* spp., 32.7% for *Bartonella* spp., 7.1% for CNM and 8.8% for *B. burgdorferi* (*s.l.*) and the prevalence rates in attached nymphs were 33.7% for *Bartonella* spp., 52.9% for *Rickettsia* spp., 13.5% for CNM and 11.3% for *B. burgdorferi* (*s.l.*) Both rodents and attached ticks were negative for *Babesia* spp. The prevalence in questing ticks was 18.2% for *Rickettsia* spp., 7.3% for CNM, 6.4% for *B. burgdorferi* (*s.l.*) and 1.4% for *Babesia* spp. All tested samples were *Anaplasma*-negative. Sequencing revealed the occurrence of 14 identified species.

**Conclusions:**

This research is the first evaluation of the prevalence for *Hepatozoon* spp. in rodents from Germany. In comparison to earlier studies, detected pathogens species remained the same; however, the prevalence for particular pathogens differed.

## Background

Small mammals are important hosts for the developmental immature stages of ticks in their natural life-cycle. Moreover, small mammals serve also as reservoirs [1] for various zoonotic agents. *Ixodes ricinus* is the most prevalent tick species in Europe and is responsible for the transmission of most zoonotic tick-borne pathogens (TBP) [2]; however, *Dermacentor reticulatus* is a rising concern as a potential vector of TBP.

*Anaplasma phagocytophilum* and “*Candidatus* Neoehrlichia mikurensis” (CNM) are Gram-negative, obligate intracellular bacteria which are tick-borne and mainly transmitted by *I. ricinus* [3]. However, *D. reticulatus* has also been described to harbour both [4, 5]. There are four ecotypes of *A. phagocytophilum* and only two are vectored by *I. ricinus* [6]. While *A. phagocytophilum* is known to cause mild to severe symptoms in humans, dogs and other mammals, CNM is rather an opportunistic agent mostly affecting immunosuppressed humans and dogs [7, 8]. CNM is considered to be harboured by rodents such as *Myodes glareolus* and *Apodemus flavicollis* [1]. Whereas roe deer, wild boars and hedgehogs are regarded as reservoirs for *A. phagocytophilum*, the reservoir function of small mammals is disputable, as there are supportive as well as refutable studies [1, 9–12].

*Rickettsia* spp. are likewise zoonotic Gram-negative, obligate intracellular bacteria which may be subdivided in four groups: (i) the spotted fever group (SFG); (ii) the typhus group; (iii) the *Rickettsia bellii* group; and (iv) the *Rickettsia canadensis* group [13]. Most rickettsiae belonging to the SFG are tick-borne and zoonotic. While *I. ricinus* is thought to be a vector in particular for *Rickettsia monacensis* and *R. helvetica*, *D. reticulatus* seems to be the main vector for *R. raoultii* in Europe [1, 13, 14]. While *R. helvetica* and *R. slovaca* are considered to be harboured by sika deer and dogs, and by wild boars and domestic ruminants, respectively, the reservoir host for *R. raoultii* is still not clear [15–17]. Nonetheless, small mammals have previously been found positive for all three aforementioned *Rickettsia* species [18, 19].

Species of the *Borrelia burgdorferi* (*sensu lato*) complex are the causative agents of Lyme disease which is the most prevalent tick-borne disease in Europe [20]. *Ixodes ricinus* is known to be the main vector and small mammals are expected to be key reservoirs for *B. afzelii* which is a species of the *B. burgdorferi* (*s.l.*) complex [21].

*Bartonella* spp. are zoonotic, Gram-negative, vector-borne bacteria. Rodents are known to be reservoirs for most *Bartonella* species [22], whilst a variety of arthropods such as fleas, lice, keds and ticks are considered to transmit these pathogens. In Germany, human cases of bartonellosis, mainly caused by *B. henselae*, have been previously reported [23].

*Babesia* spp. and *Hepatozoon* spp. are small intracellular parasites which are harboured by many different vertebrate hosts including birds and mammals in Europe [24, 25]. *Babesia microti* is mostly found in voles of the genus *Microtus*, in particular *M. agrestis* in Europe. However, there are also reports of *B. microti* in other rodent species such as *M. glareolus* and *A. flavicollis* [26]. *Ixodes ricinus* is believed to be the main vector of several *Babesia* spp. [27]. However, *I. trianguliceps*, a rodent-associated tick species, seems to be the key vector of *B. microti* in Europe. Human babesiosis caused by *B. microti* was previously reported in a human from Germany [28].

In the past, *Hepatozoon* spp. in rodents were not directly examined in Germany; however, there was an accidental finding of *Hepatozoon* sp. in one rodent previously tested by our study group [29] and other findings in *M. glareolus* and *M. oeconomus* previously from Poland, but neither in *A. flavicollis* nor insectivores [30]. Thus far the *Hepatozoon* species obtained from small mammals in Europe are either non-pathogenic or of unknown pathogenicity to humans [31]. *Hepatozoon canis*, which is highly pathogenic to dogs, was previously found in *I. ricinus* and *D. reticulatus* collected from foxes in Germany [32]. Most previous examinations on TBP in hosts and vectors from nature were carried out in a time frame of few years only and did not reassess the same areas again. Thus, long term studies on ticks, small mammals and TBP are scarce. However, it may be of importance to survey the dynamics of TBP in hosts and vectors in the purpose of predicting the distribution and maintenance of TBP in the future. Previous research showed a quite high prevalence of the previously mentioned TBPs in small mammals and ticks from Saxony, Germany [4, 18, 29, 33–36].

The present study reassessed TBPs in small mammal and tick populations from sites in Saxony which were previously examined by our group for TBP over the last 9 years [4, 18, 29, 33–36]. Thus, the aims of this study were: (i) collection of rodents, their attached ticks and questing ticks in Saxony, Germany; (ii) assessment of the prevalence of the mentioned pathogens in collected rodents and ticks; (iii) comparison of the current results with our previous studies from the last 9 years [4, 18, 29, 33–36].

## Results

### Captured rodents and their attached ticks

A total of 165 rodents belonging to four species were collected (predominantly *M. glareolus*, 63.0%, CI: 55.4–70.0%, *n *= 104; followed by *Apodemus flavicollis*, 35.8%, CI: 28.8–43.3%, *n *= 59; and two others, *A. agrarius*, *n *= 1 and *Arvicola terrestris*, *n *= 1; Table 1). Overall, 1256 ticks were attached to 122 rodents from three species (*A. agrarius*, *n *= 1; *A. flavicollis*, *n *= 42; *M. glareolus*, *n *= 79). There were only two tick species detected, *I. ricinus* (92.7%, CI: 91.1–94.0%, *n *= 1164) and *D. reticulatus* (7.3%, CI: 6.0–8.9%, *n *= 92). While *I. ricinus* parasitized on three rodent species [*A. agrarius* (*n *= 1), *A. flavicollis* (*n *= 42) and *M. glareolus* (*n *= 69)], *D. reticulatus* exclusively infested *M. glareolus* (*n *= 22). Only larvae and nymphs were observed on small mammals. Among *I. ricinus*, larvae constituted the majority **(**93.6%, CI: 92.1–94.9%, *n *= 1090), while nymphs were scarce (6.7%, CI: 5.1–7.9%, *n *= 74). However, for *D. reticulatus* the nymphs (90.2%, CI: 82.2–95.0%, *n *= 83) were more prevalent than larvae (9.8%, CI: 5.0–17.8%, *n *= 9). The maximum infestation rate on rodents was 135 ticks per host (*M. glareolus*) with a mean value of 7.6 (SD= 16.43).

**Table 1:** Numbers of collected and selected rodents, attached and questing ticks, 2015–2017, Saxony, Germany

Species	2015		2016		2017		M	Total
	M	Total	M	Total	M	Total		
Collected rodents								
*A. flavicollis*	17	17	15	15	27	27	59	59
*A. agrarius*	–	–	1	1	–	–	1	1
*A. terrestris*	1	1	–	–	–	–	1	1
*M. glareolus*	55	55	31	31	18	18	104	104
Total	73	73	47	47	45	45	165	165
Attached ticks								
*I. ricinus*	158	295	97	231	140	638	395	1164
Larvae	144	271	81	212	117	607	342	1090
Nymph	14	24	16	19	23	31	53	74
*D. reticulatus*	17	33	23	35	20	24	60	92
Larvae	3	3	–	–	6	6	9	9
Nymph	14	30	23	35	14	18	51	83
Total	175	328	120	266	160	662	455	1256
Questing ticks								
*I. ricinus*	58	299	31	42	105	206	194	547
Larvae	7	13	–	–	20	21	27	34
Nymph	25	214	2	13	50	150	77	377
Male	11	31	18	18	21	21	50	70
Female	15	41	11	11	14	14	40	66
*D. reticulatus*	–	–	5	9	21	21	26	30
Male	–	–	1	3	5	5	6	8
Female	–	–	4	6	16	16	20	22
Total	58	299	36	51	126	227	220	577

*Abbreviation*: M, selected for molecular examination

### Questing ticks

Altogether, 577 ticks belonging to two species were collected from the vegetation: *I. ricinus* was more prevalent (94.8%, CI: 92.6–96.3%, *n *= 547) than *D. reticulatus* (5.2%, CI: 3.6–7.3%, *n *= 30, Table 1). The most frequently collected developmental stage among *I. ricinus* were nymphs (68.9%, CI: 64.9–72.7%, *n *= 377), followed by adults (24.9%, CI: 21.4–28.7%, *n *= 136) and larvae (6.2%, CI: 4.5–8.6%, *n *= 34). In case of *D. reticulatus*, only adult ticks were collected and exclusively in the years 2016 and 2017 (Table 1).

### PCR results for rodents

At least 1 out of 7 tested pathogens was detected in 156 out of 165 rodents (94.5%, CI: 89.8–97.2%). None of the samples tested positive for *A. phagocytophilum* or *Babesia* spp. *Apodemus agrarius* (*n *= 1) was negative for all tested pathogens and *A. terrestris* (*n *= 1) was exclusively positive for CNM (100%, *n *= 1; Table 2). *Myodes glareolus* (*n *= 104) and *A. flavicollis* (*n *= 59) were infected with at least one of the tested pathogens at the same level, 96.2 and 93.2%, respectively (*P *= 0.462). The prevalence levels for tested pathogens differed significantly (*χ*^2^= 128.132, *df *= 4, *P <* 0.001) with *Bartonella* spp. as the most often detected pathogen (78.2%), followed by CNM (58.2%), *B. burgdorferi* (49.1%), *Rickettsia* spp. (29.1%) and *Hepatozoon* spp. (24.2%) (Table 2). Pairwise comparisons for the prevalence between the years revealed no significant differences.

**Table 2:** The prevalence of TBPs in captured rodents, 2015–2017, Saxony, Germany

Rodent species (*n*)	Prevalence of TBP (no. of positive rodents) [95% CI]				
	*Bartonella* spp.	*B. burgdorferi* (*s.l*.)	CNM	*Rickettsia* spp.	*Hepatozoon* spp.
*A. flavicollis* (*n *= 59)	78% (46) [65.7–86.8]	47.5% (28) [35.3–60]	59.3% (35) [46.6–70.9]	27.1% (16) [17.4–39.7]	6.8% (4) [2.2–16.6]
*A. agrarius* (*n *= 1)	0	0	0	0	0
*A. terrestris* (*n *= 1)	0	0	100% (1) [16.8–100]	0	0
*M. glareolus* (*n *= 104)	79.8% (83) [71–86.5]	51% (53) [41.5–60.4]	57.7% (60) [48.1–66.8]	30.8% (32) [22.7–40.2]	34.6% (36) [26.6–44.2]
Total (*n *= 165)	78.2% (129) [71.3–83.8]	49.1% (81) [41.6–56.7]	58.2% (96) [50.6–65.4]	29.1% (48) [22.7–36.5]	24.2% (40) [18.3–31.3]

All samples were negative for *A. phagocytophilum* and *Babesia* spp.

*Abbreviations*: 95% CI, 95% confidence interval; n, number of individuals

DNA of *Bartonella* spp., *B. burgdorferi* (*s.l.*) and *Rickettsia* spp. was recorded only in two rodent species, *A. flavicollis* and *M. glareolus*, with no significant differences in prevalence (*P *= 0.842, *P *= 0.745, *P *= 0.721, respectively) (Table 2). *Hepatozoon* spp. was the only pathogen which was significantly more prevalent (*P* < 0.0001) in *M. glareolus* (34.6%) than in *A. flavicollis* (6.8%). CNM was detected in three rodent species, although with no significant differences in prevalence rates regarding the rodent species (*χ*^2^= 0.754, *df *= 2, *P *= 0.686). The prevalence levels for CNM (*P *= 0.0003) and for *B. burgdorferi* (*s.l.*) (*P* < 0.0001) were significantly higher in males than in females of *M. glareolus* (77.1%, CI: 63.3–86.9%, *n *= 37 *vs* 41.1%, CI: 52.5–82.6%, *n *= 23; and 72.9%, CI: 58.9–83.5%, *n *= 35 *vs* 32.1%, CI: 21.4–45.2%, *n *= 18; respectively).

Sequencing of randomly selected rodent samples (*n *= 40; Table 3) revealed the presence of *Bartonella taylorii* (*n *= 1), uncultured *Bartonella* sp. (*n *= 5), *Hepatozoon* sp. BT-2014 isolate DB2382 (*n *= 11), *Hepatozoon* sp. clone PCE165 (*n *= 1), *R. raoultii* (*n *= 7), *R. helvetica* (*n *= 9) and *Borrelia afzelii* (*n *= 6). Co-infections in rodents (Table 4) were very common and were present in 122 small mammals (73.9%, CI: 66.7–80.1%). Triple co-infections were the most common and diverse with 9 different pathogen combinations detected in 50 rodents. The most prevalent co-infection (*n *= 25) was *Bartonella* spp. + CNM + *B. burgdorferi* (*s.l.*). Double infections with a variety of 7 different pathogen combinations were detected in 44 rodents. Three combinations of quadruple infections occurred in 18 small mammals, while the quintuple co-infections were present in 10 rodents.

**Table 3:** Sequencing results for selected samples: rodents (*n *= 40), attached (*n *= 25) and questing ticks (*n *= 23), 2015–2017, Saxony, Germany

Detected pathogen	Minimum identity (%)	GenBank ID^a^	Sample	Type	*n*
*Babesia capreoli*	100	KX839234	*D. reticulatus*	Questing	1
*Babesia microti*	99	KX591647	*I. ricinus*	Questing	1
*Babesia venatorum*	99	MG052939	*I. ricinus*	Questing	1
*Bartonella doshiae*	97	AJ269786	*I. ricinus*	Attached	1
*Bartonella grahamii*	100	CP001562	*I. ricinus*	Attached	1
			*D. reticulatus*	Attached	3
*Bartonella taylorii*	99	AJ269788	*I. ricinus*	Attached	3
			*D. reticulatus*	Attached	2
	99	AJ269784	*A. flavicollis*	–	1
*Bartonella* sp. N40	97	AJ269787	*I. ricinus*	Attached	2
			*D. reticulatus*	Attached	2
*Bartonella* sp. 15AZ DNA	99	LN847263	*I. ricinus*	Attached	1
Uncultured *Bartonella*	100	MF039571	*A. flavicollis*	–	1
	95		*M. glareolus*	–	2
	100	DQ155379	*I. ricinus*	Attached	1
	99	DQ155380	*M. glareolus*	–	2
	100	KX267692	*I. ricinus*	Attached	1
*Borrelia afzelii*	99	CP009058	*A. flavicollis*	–	1
	100	CP018262	*M. glareolus*	–	4
	100	JX971363	*M. glareolus*	–	1
*Hepatozoon* sp. BT-2014 isolate DB2382	99	KJ408528	*A. flavicollis*	–	1
			*M. glareolus*	–	10
*Hepatozoon* sp. clone PCE165	99	KX776354	*M. glareolus*	–	1
*Rickettsia raoultii*	99	CP019435	*M. glareolus*	–	1
	99	MF002527	*M. glareolus*	–	6
			*D. reticulatus*	Questing	10
*Rickettsia helvetica*	99	KU310591	*A. flavicollis*	–	2
			*M. glareolus*	–	7
			*I. ricinus*	Attached	3
			*I. ricinus*	Questing	7
	99	KT835126	*I. ricinus*	Attached	2
			*I. ricinus*	Questing	3
*Rickettsia monacensis*	100	KU961543	*I. ricinus*	Attached	1
Uncultured *Rickettsia*	95	KX591658	*I. ricinus*	Attached	1
			*D. reticulatus*	Attached	1

^a^Most similar sequence on GenBank

*Abbreviation*: n, number of samples sequenced

**Table 4:** Co-infections detected in rodent samples, 2015–2017, Saxony, Germany

No. of co-infections (*n *= 122)	Detected pathogen per co-infection					No. of rodents with co-infection
	*Bartonella* spp.	CNM	*B. burgdorferi* (*s.l.*)	*Rickettsia* spp.	*Hepatozoon* spp.	
Quintuple (*n *= 10)	+	+	+	+	+	10
Quadruple (*n *= 18)	+	+	+	+	−	9
	+	+	+	−	+	7
	+	+	−	+	+	2
Triple (*n *= 50)	+	+	+	−	−	25
	−	+	+	−	+	6
	+	+	−	+	−	5
	−	+	+	+	−	4
	+	−	+	−	+	3
	+	−	+	+	−	2
	+	−	−	+	+	2
	+	+	−	−	+	2
	−	−	+	+	+	1
Double (*n *= 44)	+	+	−	−	−	17
	+	−	+	−	−	10
	+	−	−	+	−	9
	−	+	+	−	−	3
	−	−	−	+	+	2
	+	−	−	−	+	2
	−	+	−	+	−	1

*Key*: +, presence of pathogen; −, absence of pathogen

*Abbreviation*: CNM, “*Candidatus* Neoehrlichia mikurensis”

### PCR results for attached ticks

In total, 4 out of 7 tested pathogens were detected. *Anaplasma phagocytophilum*, *Hepatozoon* spp. and *Babesia* spp. were not detected. Overall, the MIR for at least one of four detected pathogens for larvae was 62.8% (CI: 53.6–71.2%) and the general prevalence for nymphs was 75% (CI: 65.8–82.4%). However, *B. burgdorferi* (*s.l.*) was detected only in *I. ricinus* ticks, while CNM, *Bartonella* spp. and *Rickettsia* spp. were recorded in both *I. ricinus* and *D. reticulatus* (Table 5). CNM was found in *D. reticulatus* nymphs (9.8%), *I. ricinus* larvae (7.4%) and nymphs (17.4%; Table 5)*. Bartonella* spp. was detected in all examined life stages and tick species with similar prevalence rates (32–40%). *Rickettsia* spp. was significantly the most often detected pathogen in both tick species, *D. reticulatus* (73.2%; *χ*^2^= 48.963, *df *= 2, *P <* 0.001) and *I. ricinus* (46.1%; *χ*^2^= 55.312, *df *= 3, *P <* 0.001). The prevalence for *Rickettsia* spp. was significantly higher (almost 3 times) in *D. reticulatus* than *I. ricinus* concerning nymphs (*P* < 0.0001). Statistical differences in the prevalence of TBPs was noted only for *Rickettsia* spp. regarding *I. ricinus* nymphs attached to *M. glareolus* (58.3%, CI: 28.8–75.6%) and to *A. flavicollis* (3.4%, CI: 0–18.7%) (*P *= 0.0005). There were no statistical differences in the prevalence levels for different pathogens between the years, except for *Bartonella* spp. which was the highest in 2016 and the lowest in 2015 (43.7%; *χ*^2^= 6.389, *df *= 2, *P *= 0.04). Further examinations of arbitrarily selected *Rickettsia*-positive (*n *= 8) and *Bartonella*-positive (*n *= 17) samples (Table 3) revealed presence of the following species (Table 3): *R. helvetica* (*n *= 5; 5 *I. ricinus* larvae pools), *R. monacensis* (*n *= 1; 1 *I. ricinus* larvae pool), uncultured *Rickettsia* sp. (*n *= 2; 1 *I. ricinus* and 1 *D. reticulatus* larvae pools) as well as *B. grahamii* (*n *= 4; 1 *I. ricinus* and 1 *D. reticulatus* larvae pools, 2 *D. reticulatus* nymphs), *B. taylorii* (*n *= 5; 2 *I. ricinus* and 1 *D. reticulatus* larvae pools, 1 *I. ricinus* and 1 *D. reticulatus* nymphs), *B. doshiae* (*n *= 1; 1 *I. ricinus* larvae pool), *Bartonella* sp. 15AZ DNA (1 *I. ricinus* nymph), *Bartonella* sp. N40 (*n *= 4; 2 *I. ricinus* and 2 *D. reticulatus* nymphs) and uncultured *Bartonella* spp. (*n *= 2; 2 *I. ricinus* nymphs). Co-infections were only examined for nymphs as larvae samples were pooled. Out of 104 examined nymphs 29 (27.9% CI: 20.1–37.1%) were co-infected with at least 2 pathogens. There was only one pathogen combination for triple infections (CNM + *Rickettsia *+ *Bartonella*) which occurred in 6 ticks. Double infections occurred in 23 ticks with five different combinations of pathogens (15× *Rickettsia* spp. + *Bartonella* spp.; 3× *B. burgdorferi *+ *Bartonella* spp.; 3× CNM + *Bartonella* spp.; 1× CNM + *Rickettsia* spp.; and 1× *B. burgdorferi *+ CNM).

**Table 5:** The prevalence of TBPs in selected ticks attached to rodents, 2015–2017, Saxony, Germany

Tick species	No. of selected ticks (pools)	Prevalence (no. of positive ticks) [95% CI]			
		*B. burgdorferi* (*s.l.*)	*Bartonella* spp.	CNM	*Rickettsia* spp.
*I. ricinus*					
Larvae^a^	342 (108)	9.3% (10) [4.9–16.4]	32.4% (35) [24.3–41.7]	7.4% (8) [3.6–14.1]	40.7% (44) [31.9–50.2]
Nymphs	53	11.3% (6) [4.9–22.9]	32.1% (17) [21.0–45.5]	17% (9) [9.0–29.51]	28.3% (15) [17.9–41.7]
*D. reticulatus*					
Larvae^a^	9 (5)	0	40% (2) [11.6–77.1]	0	20% (1) [2.0–64.0]
Nymphs	51	0	35.3% (18) [23.6–49.1]	9.8% (5) [3.8–21.4]	78.4% (40) [65.2–87.7]

All samples tested negative for *Babesia* spp., *Hepatozoon* spp. and *Anaplasma phagocytophilum*

^a^Prevalence levels for larvae are calculated as MIR

*Abbreviations:* 95% CI, 95% confidence interval; CNM, “*Candidatus* Neoehrlichia mikurensis”

### PCR results for questing ticks

DNA of at least one of the tested pathogens was found in 63 out of 220 ticks (28.6%, CI: 23.1–35.0%). All samples were negative for *Hepatozoon* spp., *Bartonella* spp. and *A. phagocytophilum*. *Ixodes ricinus* ticks were positive for 4 out of 7 pathogens with significantly different prevalence levels (*χ*^2^= 14.841, *df *= 3, *P *= 0.002); the highest was observed for *Rickettsia* spp. (10.3%), followed by CNM (8.3%), *B. burgdorferi* (*s.l.*) (7.2%) and *Babesia* spp. (1%) (Table 6). *Dermacentor reticulatus* tested positive for only two pathogens (Table 6), with *Rickettsia* spp. (76.9%) significantly more prevalent (over 20 times) than *Babesia* spp. (3.8%) (*P* < 0.0001). The prevalence for *Rickettsia* spp. was significantly higher (almost 7.5 times) in *D. reticulatus* than in *I. ricinus* (*P* < 0.0001). The statistical difference in the prevalence rates for different pathogens between the years was noted only for *B. burgdorferi* which was the highest in 2015 in comparison to the years 2016 and 2017 (*χ*^2^= 7.363, *df *= 2, *P *= 0.03). Randomly selected *Rickettsia*-positive samples (*n *= 20) and all *Babesia-*positive samples (*n *= 3) were further sequenced (Table 3). *Rickettsia helvetica* (*n *= 10) was found in *I. ricinus*, while *R. raoultii* (*n *= 10) was found in *D. reticulatus*. Regarding *Babesia*, three species were detected: *B. capreoli* (*n *= 1) in *D. reticulatus*, and *B. microti* (*n *= 1) and *B. venatorum* (*n *= 1) in *I. ricinus*. Co-infections in questing ticks were seldom: they were present only in 8 ticks (3.6%, CI: 1.7–7.1%). Most of them occurred in *I. ricinus* (*n *= 7). Double infections were the most common (*n *= 6), with three different pathogen combinations (3× *B. burgdorferi* + *Rickettsia* spp., 2× CNM + *Rickettsia* spp. and 1× *Babesia* spp. + *Rickettsia* spp.). Triple co-infections were observed only in 2 cases: in *D. reticulatus* and *I. ricinus* ticks, with 2 different pathogen combinations (1× *B. burgdorferi *+ CNM + *Babesia* spp. and 1× *B. burgdorferi *+ CNM + *Rickettsia* spp.).

**Table 6:** The prevalence of TBPs in selected questing ticks, 2015–2017, Saxony, Germany

Tick species	Prevalence (number of positive ticks) [95% CI]			
	*Babesia* spp.	*B. burgdorferi* (*s.l.*)	CNM	*Rickettsia* spp.
*I. ricinus* (*n *= 194)	1.0% (2) [0–3.9]	7.2% (14) [4.3–11.8]	8.3% (16) [5.1–13.1]	10.3% (20) [6.7–15.5]
*D. reticulatus* (*n *= 26)	3.8% (1) [0–20.5]	0	0	76.9% (20) [57.6–89.3]
Total (*n *= 220)	1.4% (3) [0.3–4.1]	6.4% (14) [3.8–10.5]	7.3% (16) [4.5–11.6]	18.2% (40) [13.6–23.8]

*Note*: All ticks were negative for *Hepatozoon* spp., *Bartonella* spp. and *Anaplasma phagocytophilum*

*Abbreviations:* 95% CI, 95% confidence interval; CNM, “*Candidatus* Neoehrlichia mikurensis”

The prevalence for *Rickettsia* spp. was significantly higher in attached ticks in comparison to rodents and questing ticks (*χ*^2^= 40.082, *df *= 2, *P < *0.001). *Borrelia burgdorferi*, CNM, *Bartonella* spp. and *Hepatozoon* spp. were more prevalent in rodents than in questing and attached ticks (*χ*^2^= 141.338, *df *= 2, *P < *0.001; *χ*^2^= 170.022, *df *= 2, *P < *0.001; *χ*^2^= 259.132, *df *= 2, *P < *0.001; and *χ*^2^= 113.48, *df *= 2, *P < *0.001; respectively; Tables 2, 5, 6). However, 7 larvae pools/nymphs attached to uninfected rodents were positive for *Bartonella* spp.

### Comparing the present results with previous studies

The results from this study were compared with results obtained in 2009–2014 from the same sites [4, 18, 29, 33–35]. Regarding the numbers and diversity of captured small mammals, there is a visible decreasing trend. In the past, a total of 10 small mammal species were captured, while in the present study only 4 rodent species were found. Furthermore, the species of attached ticks were more diverse in the previous investigations, since *I. trianguliceps* and unidentified *Dermacentor* and *Ixodes* ticks were also found. In the present study, *A. phagocytophilum* was absent in each type of tested sample, while previously it had been detected in small mammals, questing and attached ticks [4, 29]. Rodents and attached ticks were also *Babesia*-negative, whereas before they had been positive [29, 34]. Regarding questing ticks, the prevalence for *Babesia* spp. in *I. ricinus* slightly decreased from 4.1% in 2009 to 1% in the present study (*P *= 0.0359) [29]. However, in this investigation DNA of *Babesia* was additionally found in questing *D. reticulatus.* In the present study *B. burgdorferi* (*s.l.*) was detected in questing ticks (also only in *I. ricinus*) with no statistical differences compared to the former research [33]; however, the present prevalence in small mammals (49.1%) was much higher than in the past (31.2%) (*P* < 0.0001). *Borrelia burgdorferi* (*s.l.*) in attached ticks had not been tested in the previous investigations. The prevalence for *Rickettsia* spp. in questing, attached ticks and small mammals seems to be stable over the years as it had been similar in the past [18, 33]. The infection levels of CNM seem to be increasing. The prevalence from this research was significantly higher than in the last study [4] in small mammals (41.2 *vs* 58.2%, *P *= 0.0003) and the prevalence for attached ticks in the past was fluctuating from 1.9 to 9.8% while now the average MIR for larvae was 7.1% and the average prevalence for nymphs was 13.5%. *Bartonella* spp. remained the most often detected pathogen in small mammals [35]. The prevalence in small mammals decreased from 73.9% in 2010 to 43.3% in 2013 ([35], our unpublished data] and has since (2015–2017) increased up to 78.2% (data missing for 2014). The infection levels in attached ticks also increased from 16.3% in 2010–2011 (our unpublished data) to 32.7% (MIR for larvae) and 33.7% (for nymphs) in the present study (with a gap in the years 2012–2014).

## Discussion

This study reassessed the prevalence of TBP over 9 years in ticks and rodents from sites previously examined by our group in the surroundings of Leipzig, Saxony, Germany [4, 18, 29, 33–35]. Although such long-term investigations are scarce, they may be of importance from a public health point of view to survey dynamics of TBP in hosts and vectors as this may help to predict the distribution and maintenance of TBP in future. The number of captured rodents and questing ticks as well as their species diversity has been decreasing through the years. In contrast, the average tick infestation on rodents has been rising in recent years. A reason for this phenomenon may be the so-called dilution effect. This effect describes that the higher the number of individuals in a host population the lower the tick burden per host individual [37]. In line with a former study, *D. reticulatus* was exclusively found on *M. glareolus* while *I. ricinus* did not have such a host association [18].

CNM is widespread in rodents across Eurasia with a prevalence ranging between 10.8–52.7% in Germany and other European countries, such as the Netherlands and Slovakia [36, 38, 39]. Earlier, it was described that male rodents were more often infected with CNM than females [4]. The present research confirms a sex-biased difference in the prevalence for CNM in *M. glareolus*. Previous studies explained this bias by a higher activity rate of males and due to immunosuppressive effects and higher aggression levels resulting in a higher chance of encountering the pathogen through fights [40]. Through wounds, scratches and/or bites pathogens may be transmitted directly to the bloodstream. Previous studies from Austria, France and the Netherlands showed a moderate prevalence (1.7–22%) in the known CNM vector, *I. ricinus* [41–43]. The prevalence in the present study was statistically lower in questing ticks than in previous studies [36]. CNM has been rarely investigated in *D. reticulatus* ticks. In this study it could be found only in attached *D. reticulatus* and not questing individuals, suggesting that it was probably a temporary uptake through the blood meal. Previously *B. burgdorferi* (*s.l.*) was described in rodents in other European countries with a prevalence of up to 77% in Austria [44]. In the present study, the prevalence of *B. burgdorferi* (*s.l.*) in rodents has significantly increased in the years 2015–2017 compared to 2012–2014 (from 31 to 49%) [33]. A previous investigation showed *B. burgdorferi* (*s.l.*) has many mechanisms to elude the hosts’ immune system, thus persisting in their rodent host [45]. One proven effect is described by a T-dependent B cell response which is subverted during infection in reservoir hosts. This could be a reason for the rise of the prevalence over the years. However, a dilution effect may not be ruled out as the population size of rodents decreased over the years, while the tick density increased per rodent. As described earlier for CNM, male *M. glareolus* were likewise more often infected than females. Sequencing from rodent samples confirmed the presence of pathogenic *B. afzelii*, the main rodent-associated *Borrelia* species [46]. Although the prevalence in small mammals increased, it did not vary in ticks over the years in this study. The prevalence in questing and attached *I. ricinus* ticks from the present study was in line with other European countries, e.g. Estonia, Belarus, Slovakia and Austria (8.2–13.5%) [14, 47, 48]. *Rickettsia* spp. were found in almost 24% of the rodents from this study which was higher compared to the prevalence detected in other parts of Germany, e.g. Mecklenburg-Western Pomerania, Thuringia and Baden-Wuerttemberg (6.8–9.4%) [49], and similar to a study from Lithuania (27.6%) [50]. Previous investigations in Europe revealed the occurrence of *R. helvetica* in *A. agrarius*, *A. flavicollis* and *M. glareolus* [51]. An earlier study by our group also showed the presence of *R. raoultii* in small mammals [18]. DNA of *Rickettsia* spp. was found in larvae attached to positive as well as to negative rodents, which supports the hypothesis of transovarial transmission of *Rickettsia* in ticks [52]. The current prevalence of 10.3% in *I. ricinus* is relatively low compared to the prevalence from previous studies in Germany (18–25%) and other European countries, e.g. France (16%) [18, 33, 53]. The infection level in attached (20–78.4%) and questing (76.9%) *D. reticulatus* ticks from the present study was much higher than in *Dermacentor* ticks from Poland and the Czech Republic (18–41%) [54, 55]. The previous prevalence from the same sites showed a likewise high prevalence in questing *D. reticulatus* (70.5%) [33]. *Rickettsia raoultii* was detected only in questing *D. reticulatus* ticks with a very high prevalence and in *M. glareolus* with a low infection rate, which is in accordance with studies suggesting the transovarial transmission of *R. raoultii* in *D. reticulatus* is more significant than feeding on reservoir hosts in order to maintain in the natural life-cycle [18]. *Bartonella* spp. in rodents are highly prevalent in Europe with prevalence rates ranging between 16–56% in France, Denmark and Poland [56–58]. In the present study the prevalence was 78% in rodents and thus the highest in comparison to all other examined TBPs. A previous examination at the same study sites [35] detected a lower prevalence of 65.8% and the following species: *B. grahamii*, *B. taylorii*, *Bartonella* sp. N40; and a variety of uncultured *Bartonella* species. In the present study, only *B. taylorii* and uncultured *Bartonella* strains were detected. *Bartonella taylorii* is known to be non-pathogenic to humans and the uncultured *Bartonella* spp. are currently of unknown pathogenicity [59]. Previously it had been shown that the prevalence for *Bartonella* spp. is significantly higher in *Apodemus* than in *Myodes* due to a deficiency in resolving the infection in *Apodemus* [60]. However, it was also shown that the prevalence of *Bartonella* spp. in *M. glareolus*, studied over 11 years, was subjected to great fluctuations and may even double over the years before declining again, as the prevalence is dependent on changes in the rodent population such as density and mean age [61]. *Bartonella* spp. could not be detected in questing ticks from the present study, supporting the hypothesis that ticks play a subordinate role in the transmission of rodent-associated *Bartonella*. Earlier studies from our group, however, support the hypothesis that ticks play a role in the life-cycle of *Bartonella* spp. since *B. chomelii* was detected in ticks attached to rodents. This *Bartonella* species is, however, associated with domesticated ruminants [62]. In the present study, seven attached larvae pools/nymphs were positive for *Bartonella* spp. even though the host was negative. Previously our group suggested that *D. reticulatus* plays a subordinate role in the transmission cycle compared to *I. ricinus*. However, the present study found almost equally high prevalence rates in attached *D. reticulatus* and *I. ricinus*. To our knowledge, there are thus far no studies focussed on the presence of *Hepatozoon* spp. small mammals in Germany. Studies from Spain, Slovakia and Poland reported a prevalence range of 4.5–41.6% in different rodent species, including *A. flavicollis* and *M. glareolus* [30, 63, 64]. In the present study, the prevalence of *Hepatozoon* spp. in rodents was 31.1%. In accordance with a study from Slovakia, *M. glareolus* showed a significantly higher prevalence than *A. flavicollis* [64]. This was also observed in rodents from Finland and Poland [30, 65]. *Hepatozoon* strains detected in small mammals from this study are known to have a wide host range and were previously detected in small mammals and reptiles [66]. It is not surprising that attached ticks as well as questing ticks were negative for *Hepatozoon* spp. in the present study, as rodent-associated *Hepatozoon* spp. are mainly transmitted by rodent-associated fleas [67]. *Babesia* DNA in this research was barely detected in questing ticks (1.4%) and not at all in rodents or in attached ticks. However, previous investigations from the same study sites revealed a similar prevalence in questing ticks (1.6%) and very low prevalence in attached ticks (0.3–0.5%) and rodents (0.6–2.5%) [29, 34]. The prevalence for *Babesia* in rodents from other European studies showed similarly low levels in rodents; however, the study from the UK reported a much higher prevalence (27.2%) [68]. The prevalence in questing ticks in former studies from Sweden and Poland varied but also in a lower range (up to 4.6%; *B. venatorum*, *B. microti* and *B. divergens*) [69, 70]. In the present study, *B. venatorum* and *B. microti* were detected in *I. ricinus*, and *B. capreoli* in *D. reticulatus*. *Babesia venatorum* and *B. microti* are zoonotic agents and have been previously detected in *I. ricinus* from other European countries [69–71]. Thus far only the “Jena” strain of *B. microti* is thought to be pathogenic to humans in Europe [72]. However, the *B. microti* strain detected in this study showed 99% identity to a non-pathogenic Ukrainian *B. microti* strain. *Babesia capreoli*, which is thought to be non-pathogenic, has previously been described in *I. ricinus*, with reindeer serving as main hosts in Europe [71, 73]. Interestingly, exclusively these three *Babesia* species described here were also previously detected at the same study sites [29].

In other studies from Germany, the prevalence for *A. phagocytophilum* in ticks varied between 1.9–8.9% [74–76]. In this investigation *A. phagocytophilum* DNA was neither detected in rodents nor in ticks. However, earlier results from our group showed a low prevalence in both rodents (1.1%; [4]) and questing ticks (5.3%; [29]). The explanation for the observed decline may be the effect of the resistance against *A. phagocytophilum* developed by rodents which may persist from 12 weeks up to a year, protecting them from re-infection and preventing uninfected ticks from infection, thus interrupting the infection cycle [77].

In comparison to the overall prevalence for TBPs in attached and questing ticks from this study, the level for rodents was generally higher, also resulting in a high co-infection rate. Even though the co-infection levels in questing ticks were very low as well as the prevalence for *Babesia* spp. (only 3 out of 220 ticks), most *Babesia*-positive ticks were co-infected leading to the assumption that infections with *Babesia* favour co-infections with other pathogens. The current co-infection level in rodents (over 70%) is much higher compared to a study from Austria where only 8.1% of the rodents were infected with more than one pathogen [78].

## Conclusions

This study reports very high prevalence levels for TBP, especially in rodents. This is the first study focussing on the presence of *Hepatozoon* spp. in rodents from Germany. Furthermore, over a 9-year long trend, it should be taken into account that the number and the species diversity of rodents and questing ticks collected have been declining, while the average infestation rate for ticks attached to rodents has been increasing. While prevalence for *A. phagocytophilum* and *Babesia* spp. in general decreased or/and were not detected at all in the present study, the prevalence for CNM, *Bartonella* spp. and *B. burgdorferi* (*s.l.*), particularly in rodents, seems to be rising. *Rickettsia* spp. are the only pathogens in which the prevalence in rodents, attached and questing ticks has remained at the same level over the years. Although the prevalence rates for certain pathogens differed between the years, the detected pathogen species did not change with time.

## Methods

### Collection sites

Rodents and questing ticks were sampled from 2015 to 2017 at four locations in the surroundings of Leipzig, Saxony, Germany. The sites had previously been described, examined and named (“E”, “F”, “H1” and “H2”) [35]. Sites E (51°15′36.5″N, 12°21′00.4″E) and F (51°17′00.9″N, 12°21′02.8″E) are located in the east and the north of the lake “Cospuden” which was artificially created from a former brown coal mining area. Site H1 (51°18′14.6″N, 12°24′41.4″E) and H2 (51°17′35.5″N, 12°24′07.5″E) are also renatured areas and parts of the “Lößnig-Dölitz” city park which is also a renatured site and was created on a former waste disposal area.

### Small mammal trapping

The trapping of small mammals took place in April to October 2015, May to November 2016 and March to October 2017. Twenty-five Sherman© live animal traps (H. B. Sherman Traps Inc., Tallahassee, FL, USA) were set for two successive nights each month at each site at the same time. Apple slices were used as bait and hay as isolation material. The traps were controlled twice a day; captured rodents were anesthetized on the spot with CO_2_ and euthanized by cervical dislocation. The rodents were morphologically identified using taxonomic key [79] and dissected in the laboratory. Attached ticks, skin and spleen samples were taken from each rodent and stored at -80 °C until further processing.

### Attached and questing ticks

Questing ticks were collected simultaneously with each rodent trapping action by the flagging method. Questing and attached ticks were stored at −80 °C until morphological identification [80] and further analysis. A total of 455 ticks were selected for further PCR analysis examining tick-borne pathogens, including 231 *I. ricinus* (207 larvae and 24 nymphs) obtained from 64 *M. glareolus*, 164 *I. ricinus* (135 larvae and 29 nymphs) from 41 *A. flavicollis* and 60 *D. reticulatus* (9 larvae, 51 nymphs) from 15 *M. glareolus* (Table 1). Altogether 351 larvae were tested in 113 pools: 342 *I. ricinus* larvae in 108 pools and 9 *D. reticulatus* larvae in 5 pools. Concerning questing ticks, a total of 194 *I. ricinus* and 26 *D. reticulatus* were selected for further molecular examination.

### DNA extraction from rodents and ticks

For DNA extraction, 0.6 g of sterile ceramic beads (sized 1.4 mm, Peqlab Biotechnologie, Erlangen, Germany) and 500 μl of PBS were added to each rodent sample. For ticks, 1 g of steel beads (sized 2.8 mm) was used instead of ceramic beads. The samples were then homogenized at 5500× *rpm* for 3 × 15 s with 10 s break intervals in a Precellys®24 tissue homogenizer (Bertin Technologies, Montigny Le Bretonneux, France). Due to financial restrictions, not all ticks were selected for further analysis. Up to five questing ticks per tick species, collection site, per month and year were randomly chosen. Attached ticks were likewise selected with the addition of up to five attached specimens per small mammal species (up to 30 host individuals per rodent species per month and collection site). Attached larvae were further tested in pools of up to 5 individuals according to the selection criteria. DNA was extracted with a QIAamp DNA Mini Kit (Qiagen, Hilden, Germany) as per the manufacturer’s recommended protocol, followed by quantitative and qualitative measures with a spectrophotometer (NanoDrop® 2000c, Thermo Fisher Scientific, Waltham, Ma, USA).

### PCR methods

All DNA samples were screened for the presence of *A. phagocytophilum*, *Babesia* spp., *B. burgdorferi* (*s.l.*), CNM and *Rickettsia* spp. by real-time and/or conventional PCRs. Samples positive for *B. burgdorferi* (*s.l.*) were additionally processed *via* multi locus sequence typing (MLST). All samples were moreover examined for *Bartonella* spp. and *Hepatozoon* spp. Details on used PCR protocols are presented in Table 7. For the detection of *Hepatozoon* spp., the initial annealing was changed to 52 °C. All *Babesia*-positive samples (*n *= 3) and a randomly selected number of samples positive for *Bartonella* spp. (*n *= 23), *Hepatozoon* spp. (*n *= 12), *Borrelia* spp. (*n *= 6) and *Rickettsia* spp. (*n *= 44; Table 3) were commercially sequenced (Interdisziplinäres Zentrum für Klinische Forschung, Leipzig, Germany). Results were aligned using Bionumerics v.7.6.1 (Applied Maths Inc., Austin, TX, USA) and compared with sequences published in GenBank using BLASTn. New allelic combinations were registered in the *Borrelia* spp. MLST database under the sequence types ST 787–792.

**Table 7:** Details on primers and PCR assays used for the detection of tick-borne pathogens in different tissues from rodents and ticks

Pathogen	PCR type	Primer name	Primer/probe sequence (5′–3′)	Gene (amplicon size) (bp)	Reference
*Anaplasma phagocytophilum*	RT	ApMsp2f	ATGGAAGGTAGTGTTGGTTATGGTATT	msp2 (77)	[81]
		ApMsp2r	TTGGTCTTGAAGCGCTCGTA		
		ApMsp2p	FAM-TGGTGCCAGGGTTGA GCTTGAGATTG-BHQ1		
*Babesia* spp.	C	BJ1	GTCTTGTAATTGGAATGATGG	*18S* rRNA (411–452)	[82]
		BN2	TAGTTTATGGTTAGGACTACG		
*Bartonella* spp.	C	Ba325s	CTTCAGATGATGATCCCA AGCCTTCTGGCG	16S-23S rRNA (ITS) (453–780)	[83]
		Ba1100as	GAACCGACGACCCCCTGCTTGCAAAGC		
*Borrelia burgdorferi* (*s.l.*)	RT	FlaF1a	AGCAAATTTAGGTGCTTTCCAA	*P41* (96)	[84]
		FlaR1	GCAATCATTGCCATTGCAGA		
		FlaProbe1	FAM-TGCTACAACCTCATCTG TCATTGTAGCATCTTTTATTTG-BBQ		
	MLST^a^	cplAF1255	AAAGATAGATTTCTTCCAGAC	*clpA* (579)	[33, 85]
		cplAR2104	GAATTTCATCTATTAAAAGCTTTC		
		clpXF403	GCTGCAGAGATGAATGTGCC	*clpX* (624)	
		clpXR1124	GATTGATTTCATATAACTCTTTTG		
		nifF1	ATGGATTTCAAACAAATAAAAAG	*nifS* (564)	
		nifR719	GATATTATTGAATTTCTTTTAAG		
		pepXF449	TTATTCCAAACCTTGCAATCC	*pepX* (570)	
		pepXR1115	GTTCCAATGTCAATAGTTTC		
		pyrF448	GATTGCAAGTTCTGAGAATA	*pyrG* (603)	
		pyrR1154	CAAACATTACGAGCAAATTC		
		recF917	CCCTTGTTGCCTTGCTTTC	*recG* (651)	
		recR1658	GAAAGTCCAAAACGCTCAG		
		rplfF40	TGGGTATTAAGACTTATAAGC	*rplB* (624)	
		rplR760	GCTGTCCCCAAGGAGACA		
		uvrF1434	GAAATTTTAAAGGAAATTAAAAGTAG	*uvrA* (570)	
		uvrR2111	CAAGGAACAAAAACATCTGG		
“*Candidatus* Neoehrlichia mikurensis”	RT	NMikGroEL F2	CCTTGAAAATATAGCAAGATCAGGTAG	*groEL* (99)	[36, 38]
		NMikGroEL rev1	CCACCACGTAACTTATTTAGCACTAAAG		
		NMikGroEL rev2	CCACCACGTAACTTATTTAGTACTAAAG		
		NMikGroEL-P2a	FAM-CCTCTACTAATTATTGCT GAAGATGTAGAAGGTGAAGC-BHQ1		
*Hepatozoon* spp.	C	Hep-1f	CGCGAAATTACCCAATT	*18S* rRNA (660)	[63, 86]
		Hep-2r	CAGACCGGTTACTTTYAGCAG		
*Rickettsia* spp.	RT	Pan Rick gltA_2 for	ATAGGACAACCGTTTATTT	*gltA* (70)	[87]
		Pan Rick gltA_2 rev	CAAACATCATATGCAGAAA		
		Pan Rick gltA_3 taq	6FAM-CCTGATAATTCGTTA GATTTTACCG–TMR		
	C^a^	120–2788	AAACAATAATCAAGGTACTGT	*OmpB* (811)	[88]
		120–3599	TACTTCCGGTTACAGCAAAGT		

^a^Only carried out if real-time PCR yielded

*Abbreviations*: C, conventional PCR; RT, real-time PCR; MLST, multi-locus sequence typing

### Statistical analysis

Confidence intervals (95% CI) for the prevalence of pathogens were determined by the modified Wald method using GraphPad Prism v.4 (Graph Pad Software, San Diego, CA, USA). Chi-square and Fisher’s tests were used to test the prevalence levels for significant independence. The significance threshold was set at *P *= 0.05. The prevalence levels for attached larvae are given as MIR (minimal infection rate) as these were pooled.

## Abbreviations

BLAST: Basic Local Alignment Search Tool
CI: confidence interval
CNM: “*Candidatus* Neoehrlichia mikurensis”
ITS: intergenic spacer
MIR: minimum infection rate
MLST: multi-locus sequence typing
PBS: phosphate-buffered saline
PCR: polymerase chain reaction
SD: standard deviation
SFG: spotted fever group
ST: sequence type
TBP: tick-borne pathogens
